# Opioid drug use in emergency and adverse outcomes among patients with chronic obstructive pulmonary disease: a multicenter observational study

**DOI:** 10.1038/s41598-020-61887-2

**Published:** 2020-03-19

**Authors:** Damien Viglino, Raoul Daoust, Sebastien Bailly, Caroline Faivre-Pierret, Isma Charif, Matthieu Roustit, Jean Paquet, Guillaume Debaty, Jean-Louis Pépin, Maxime Maignan, Jean-Marc Chauny

**Affiliations:** 1 Emergency Department and Mobile Intensive Care Unit, Grenoble Alpes University Hospital, Grenoble, France; 2 INSERM U1042, HP2 Laboratory, Grenoble-Alpes University, Grenoble, France; 3 Department of Emergency Medicine, Research Centre, Sacré-Coeur Hospital of Montreal, Montreal, Quebec Canada; 4 Department of Physiology and Sleep, Grenoble Alpes University Hospital, Grenoble, France; 5 Clinical Pharmacology Department, INSERM CIC1406, Grenoble Alpes University Hospital, Grenoble, France

**Keywords:** Chronic obstructive pulmonary disease, Adverse effects

## Abstract

There is still debate as to the safety of non-palliative opioid administration to chronic obstructive pulmonary disease (COPD) patients punctually treated for an acute complaint. All patients over 40 presenting at two university hospital emergency departments (Montréal Qc, Grenoble Fr) from March 2008 to September 2014 with dyspnea, abdominal pain or trauma were retrieved, and COPD patients were selected. Our primary endpoint was a composite criterion including invasive ventilation and death. Comparisons between visits in which opioid drugs were prescribed and those without opioids were performed using an inverse probability of treatment and censoring weight (IPTCW) estimator to adjust for baseline confounders. A survival weighted Cox model was used. 7799 visits by COPD patients were identified, corresponding to 4173 unique patients. Opioid drug prescription was reported in 1317 (16.9%) visits. After applying IPCTW weighting, opioid prescription was found to be associated with the composite criterion of poor clinical outcomes (HR = 4.73 (2.94; 7.61), p < 0.01). When taken separately, this association remained significant for invasive ventilation and death, but not for NIV. All sensitivity analyses confirmed the association, except for patients with trauma or abdominal pain as the main complaint. This excess risk is observed whatever the route of administration.

## Introduction

Pain is a common complaint in patients with chronic obstructive pulmonary disease (COPD), and is reported by 30 to 60% of patients with mild to severe COPD^1,2^, with a deleterious impact on their health-related quality of life^3–7^. This results in the high consumption of pain-related medications, including short- and long-acting opioids, and in a substantial increase in healthcare costs^8^. Recent pharmacoepidemiologic data show that opioids are commonly prescribed in COPD, with almost 50% of patients receiving oral analgesics containing opioids^9,10^. However, the safety of non-palliative opioid drug use in this population remains questionable. The prescription of opioids for chronic pain in COPD patients has been reported as triggering respiratory adverse events, especially in elderly COPD patients^11^, and when combined with benzodiazepines^12^.

To date, there is no data on the safety of opioid drug use for COPD patients in acute settings such as emergency departments (ED) or intensive care units (ICU). Opioid prescription may be challenging because of the fear of triggering opioid induced respiratory depression^13^, or dependence^14,15^. Nonetheless, the prescription of opioids may be necessary for acute pain or increased dyspnea, outside palliative situations. In this study, we sought to determine if non-palliative opioid drug administration for acute complaints in a COPD population was associated with poor clinical outcomes.

## Methods

### Study design and setting

Data were extracted from the databases of two university hospital ED with more than 50,000 patient visits per year (Sacré-Coeur de Montréal Hospital, Quebec, Canada and Grenoble Alpes University Hospital, France). Both are level 1 trauma centers and are local reference centers for chest and respiratory diseases. The databases consist of detailed electronic medical records of patients attending the ED including demographic data, medical records with timestamped treatments, final diagnosis and outcomes such as ICU admission, and in-hospital death. Institutional review board approval was obtained by each center (University of Montreal, Quebec, Canada; and CECIC Rhône-Alpes-Auvergne, Clermont-Ferrand, France IRB N°5891). Methods and data collection were conducted in accordance with guidelines for retrospective research.

### Selection of participants

All patients over 40 years of age who presented at the participating EDs for dyspnea, abdominal pain or trauma from March 16, 2008 to September 28, 2014 were initially included. Patients with a decision for palliative care (recorded intervention of the palliative care team or documented multidisciplinary procedure for limitation of care) were excluded. Each visit was considered as an independent event, and a given patient could have been entered more than once in the database if she/he had consulted the ED several times during the study period. Data were anonymized with each patient being assigned a unique number. Then COPD patients were detected using the International Classification of Diseases - tenth revision (ICD-10) codes related to COPD: simple chronic bronchitis (J41); unspecified chronic bronchitis (J42); emphysema (J43); other chronic obstructive pulmonary disease (J44); interstitial emphysema (J98.2); and compensatory emphysema (J98.3). In addition to this selection, a text mining procedure was used to detect medical records containing the term “COPD” or equivalent in the patient’s diagnosis or medical history. To confirm the detection of COPD patients, we calculated the Cohen-Kappa coefficient of agreement between two methods of COPD patient selection: ICD and text mining vs. manual selection of cases by a board-certified physician. For a target Kappa of 0.8 [0.7–0.9] and a COPD prevalence of 0.06, 848 electronic medical records needed to be reviewed^16^, giving a Cohen-Kappa coefficient of 0.92 [0.87–0.96].

### Methods and measurements

The demographic and clinical data collected included: age, gender, means of transport to the ED, vital signs at presentation, and triage level (a 5 level scale both in France and Quebec, 1 being the most urgent and 5 the least urgent).

The following molecules were considered as opioids: morphine, hydromorphone, oxycodone, meperidine, fentanyl, and sufentanil. The date and time(s) of opioid administration(s) were recorded and all events timestamped. In order to exclude opioids administered for sedation during intubation as the cause of need for ventilatory support, we eliminated opioid administrations made within 15 minutes before such procedures.

The primary endpoint was a composite criterion including invasive ventilation and death. Only death whilst in the ED, ICU or within 48 hours after hospital admission was considered for the primary endpoint. Secondary endpoints were the following events taken individually: invasive ventilation, death, ICU admission, non-invasive ventilation (NIV) and naloxone use.

Subgroup analyses were performed according to the patients’ main complaint (dyspnea, abdominal pain, or trauma). Finally, we performed sensitivity analyses taking into account (1) only the first visit in cases of multiple emergency room visits; (2) the risk associated with a parenteral administration of opioids; and (3) the risk associated with an enteral administration of opioids.

### Analysis

To account for missing data multiple imputations were performed using a fully conditional specification method with logistic regression for categorical variables and linear regression for continuous variables. Twenty imputed datasets were created to consider variables with less than 20% of missing values.

A descriptive analysis of the patients’ characteristics was performed using median and interquartile range (IQR) for quantitative data, and frequencies and percent for qualitative data. The percentage of patients with multiple visits was calculated. The characteristics of the two groups (opioids vs no-opioids) were compared using the Chi-squared test for qualitative data and Mann–Whitney test for quantitative data. To estimate the effect of opioid administration on the composite outcome, a multivariate survival Cox model using an inverse probability of treatment and censoring weight (IPTCW) estimator with a robust estimation of the variance was used. These models have been extensively described elsewhere^17,18^. Briefly, the four steps were: first, a univariable analysis of baseline variables associated with opioid administration and the composite outcome was performed to identify variables with p values <0.20. Second, these variables were introduced in a non-parsimonious multivariable logistic regression model to compute the inverse probability of treatment weights (IPTW) for individual patients. Steps one and two assessed the probability of being discharged alive before 48 hours and to compute the inverse probability of censoring weights (IPCW). Third, The IPTW and IPCW were stabilized by multiplying the weights by the probability of observing the events (opioid administration for IPTW or discharge alive for IPCW) in the overall population and the IPTCW computed by multiplying IPTW and IPCW. To avoid an over-dispersion of the weights, they were truncated at the 1^st^ and the 99^th^ percentiles^17^. A mean stabilized weight (IPTCW) of one was considered to exclude violation of the positivity assumption. Fourth, a multivariable Cox model, using the IPTCW, was used to assess the risk of opioid administration with respect to the composite outcome. To account for the delay in opioid administration after admission to the ED, a variable “opioid administration” was introduced as a time-dependent variable in the weighted Cox model. Variables with p values <0.2 were introduced in the multivariable model using a stepwise selection method to obtain the final multivariable model. Centre and period effects were introduced as fixed effects in the multivariable model.

The same methodology was used for the secondary outcomes. Statistical analyses were performed using SAS v9.4 (SAS Institute Inc., Cary, NC, USA). A p value less than 0.05 was considered significant.

### Ethics approval and consent to participate

Institutional review board approval was obtained by each center (University of Montreal, Quebec, Canada; and CECIC Rhône-Alpes-Auvergne, Clermont-Ferrand, France IRB 5891). Patient informed consent was waived given the large retrospective database design of the study, and approved by the institutional review boards and the French National commission on data protection and freedoms (declaration CNIL N°1987785v0).

## Results

### Characteristics of study subjects

**Figure 1:**
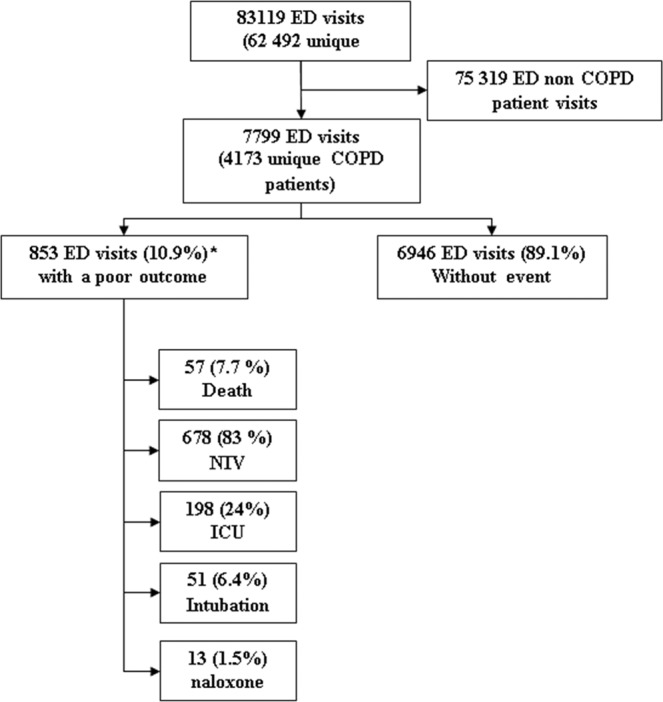
Selection Flow chart. *ED:* Emergency department *An ED visit can give rise to several outcomes.

A total of 83,119 emergency visits for dyspnea, abdominal pain or trauma were screened (Fig. 1) of which 7,799 ED visits by COPD patients (corresponding to 4,173 unique patients) were identified. In 1,317 (16.5%) of these visits, an opioid was prescribed. The epidemiological and clinical descriptions of this population are shown in Table 1.

**Table 1:** COPD population-related visit characteristics.

	Without opioids (n = 6,482 visits)	With opioids (n = 1,317 visits)	p value	Missing n (%)
Age, years	78 (69–84)	75 (65–83)	<0.01	0 (0)
Male, n (%)	3,476 (53.6)	624 (47.4)	<0.01	0 (0)
**Visit characteristics**				
Main Complaint				0 (0)
Dyspnea, n (%)	4,646 (71.7)	620 (47.1)	<0.01	
Abdominal pain, n (%)	915 (14.1)	327 (24.8)	<0.01	
Trauma, n (%)	921 (14.2)	370 (28.1)	<0.01	
Ambulance transport, n (%)	4,722 (72.9)	1,038 (78.9)	<0.01	5 (0)
Emergency triage level				830 (10.6)
1–2, n (%)	1,848 (32.3)	461 (36.9)	<0.01	
3, n (%)	3,195 (55.8)	658 (52.7)	<0.01	
4 or 5, n (%)	678 (11.9)	129 (10.3)	<0.01	
**Clinical characteristics at admission**				
GCS	15 (15–15)	15 (15–15)	0.64	6,035 (77.4)
Heart rate, /min	90 (77–103)	89 (77.5–102)	0.71	731 (9.4)
MAP, mmHg	94 (84–106)	94 (84–106)	0.42	761 (9.8)
RR, /min	24 (20–28)	20 (17–26)	<0.01	2,441 (31.3)
Pulse oximetry, %	96 (93–98)	96 (94–98)	0.06	837 (10.7)
Temperature, °C	36.7 (36.2–37.2)	36.6 (36.1–37.1)	<0.01	2,530 (32.4)
VAS > 60 mm, n (%)	1,018 (19.2)	840 (69.4)	<0.01	1,291 (16.6)

Numerical data are presented as median (interquartile range).

GCS: Glasgow coma scale, MAP: mean arterial pressure, RR: respiratory rate, VAS: visual analogue scale.

We observed an increase in the prescription of non-palliative opioids for COPD patients during the 6.5 years of the study (13.8% to 22.2%, p < 0.01). The molecules administered were morphine (n = 618, 47%); oxycodone (n = 307, 23.3%); hydromorphone (n = 220, 16.7%); fentanyl (n = 169, 12.8%) and meperidine (n = 3, 0.2%); and 56.6% of opioid administrations were made by a parenteral route. Only analgesic purpose was observed, whatever the subgroup observed: abdominal pain, trauma or dyspnea. Among the 618 patients receiving morphine, 558 (90%) were IV titration with a target dose-weight of 0.1 mg/kg (or >4 mg), and 60 (10%) with lower doses.

Among the 307 patients receiving oxycodone, 290 (94%) received 5 mg per os, 15 (5%) 10 mg, and 2 (1%) >10 mg. Among the 220 patients receiving hydromorphone, 173 were per os (123 from 1 to 3 mg and 50 with >3 mg doses) and 47 were IV titrations.

Among the 108 (1.3%) visits associated with the composite primary endpoint, 51 (47%) required invasive ventilation and 57 (53%) of patients died before 48 hours. Overall, 853 (10.9%) visits were associated with a poor outcome including ICU admission, non-invasive ventilation and naloxone use (Fig. 1).

### Main results

**Figure 2:**
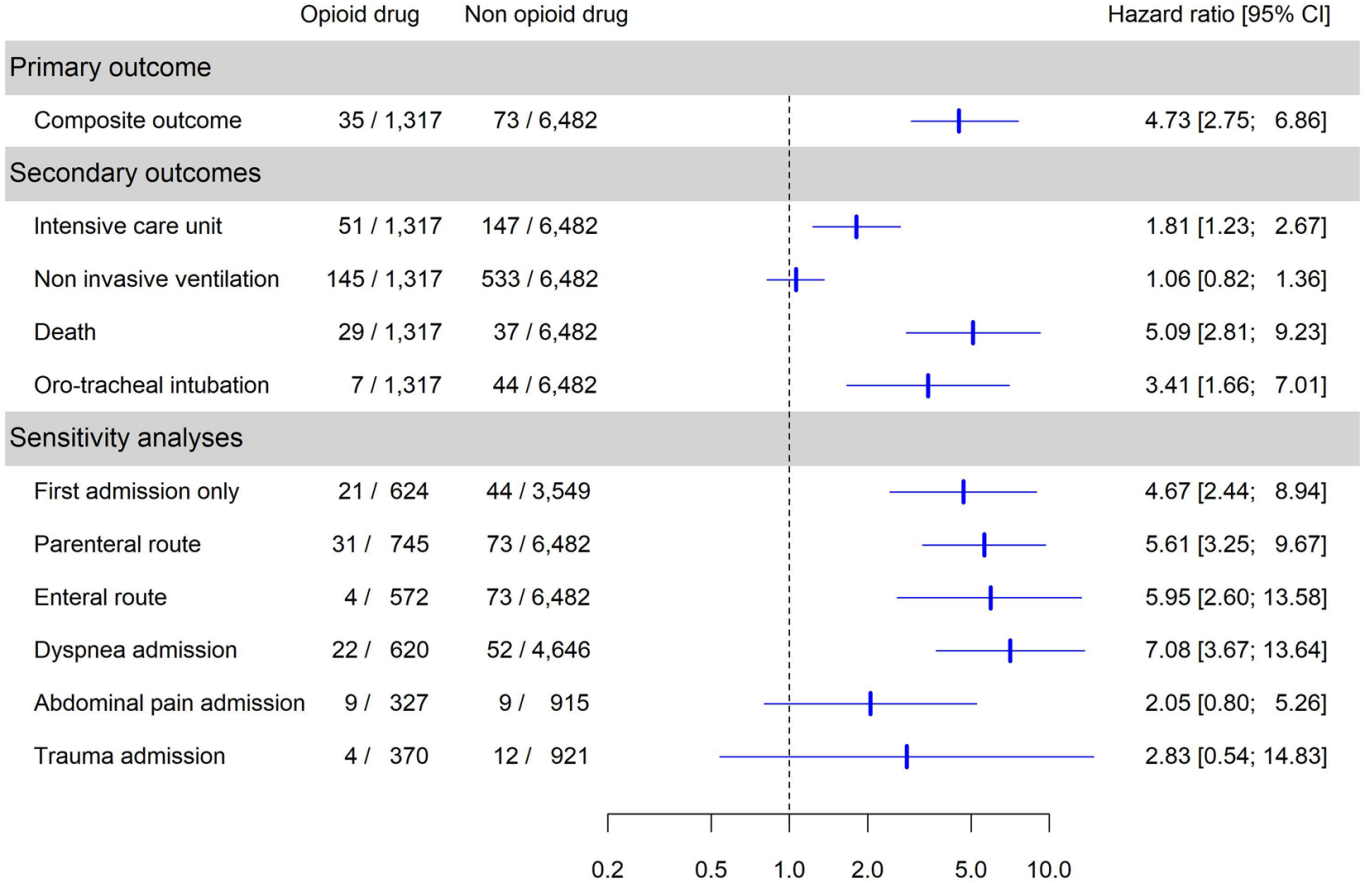
Odd ratio’s Forrest plot of primary and secondary outcomes.

The following variables were retained to compute individual weights for opioid administration (IPTW): age, year, center of inclusion, triage level, means of transport and SaO_2_ by pulse oximetry. For the censored weight (IPCW), the same variables were retained. In a multivariable Cox model, after IPTCW weighting, opioid administration to patients with COPD was significantly associated with the principal composite endpoint (Hazard ratio (HR) = 4.73 (2.94; 7.61), p < 0.01, Table 2). Separately, we observed an association between opioid use and ICU admission (HR = 1.81 (1.23; 2.67), p < 0.01), death (HR = 5.09 (2.81; 9.23), p < 0.01), and invasive ventilation (HR = 3.41 (1.66; 7.01), p < 0.01), but not with NIV (HR = 1.06 (0.82; 1.36), p = 0.65). Analysis was not performed for the association between naloxone use and opioid administration because we collected only 13 cases. In sensitivity analyses where only the first visit by patients with multiple visits was included, the same association was observed between opioids and the principal composite endpoint (HR = 4.67 (2.44; 8.94), p < 0.01, Table 3). When we considered only patients treated by a parenteral route, they presented more composite criteria events than patients not treated with opioids (HR = 5.61 (3.25; 9.67), p < 0.01). The enteral route also remained associated with poor outcomes (HR = 5.95 (2.60; 13.58), p < 0.01). The main results are summarized in Fig. 2.

**Table 2:** Multivariable analysis of the association between prescription of opioids to COPD patients and primary composite outcome: 48-hour Intubation or Death.

Variable	Class	Multivariable HR (95%CI)	p value
Age (years)	<56	ref.	ref.
	56–65	2.17 (0.53; 8.90)	0.28
	65–80	1.97 (0.53; 7.31)	0.31
	>=80	1.81 (0.49; 6.72)	0.38
Year of inclusion	2008	0.79 (0.26; 2.38)	0.68
	2009	0.92 (0.36; 2.33)	0.86
	2010	1.18 (0.50; 2.76)	0.70
	2011	1.20 (0.53; 2.71)	0.66
	2012	0.92 (0.40; 2.09)	0.84
	2013	1.07 (0.48; 2.38)	0.87
	2014	ref.	ref.
Center of inclusion	Montreal	ref.	ref.
	Grenoble	2.79 (1.69; 4.62)	<0.0001
Pulse oximetry (%)	<92	1.22 (0.66; 2.26)	0.52
Means of transport	ambulance	1.58 (0.86; 2.89)	0.14
	no ambulance	ref.	ref.
Triage level	1 or 2	1.82 (1.12; 2.94)	0.02
	3 or 4 or 5	ref.	ref.
**Opioid treatment**	**(yes)**	**4.73 (2.94; 7.61)**	**<0.0001**

HR: Hazard ratio.

**Table 3:** Multivariable analysis of the association between prescription of opioids to COPD patients and the composite outcome (48-hour Intubation or Death) taking into account only the first visit.

Variable	Class	Multivariate HR (95%CI)	p value
Age (years)	<56	ref.	ref.
	>=80	0.86 (0.46; 1.58)	0.62
Year of inclusion	2008	0.75 (0.18; 3.08)	0.69
	2009	1.16 (0.33; 4.06)	0.82
	2010	0.90 (0.24; 3.37)	0.88
	2011	1.07 (0.31; 3.74)	0.92
	2012	1.13 (0.34; 3.79)	0.84
	2013	1.09 (0.32; 3.73)	0.89
	2014	ref.	ref.
Center of inclusion	Montreal	ref.	ref.
	Grenoble	2.51 (1.31; 4.82)	<0.01
Pulse oximetry (%)	<92	1.73 (0.81; 3.73)	0.16
Means of transport	ambulance	2.23 (0.93; 5.35)	0.07
	no ambulance	ref.	ref.
Triage level	1or 2	1.94 (1; 3.77)	0.05
	3 or 4 or 5	ref.	ref.
**Opioid treatment**	**(yes)**	**4.67 (2.44; 8.94)**	**<0.01**

For the 5267 visits when patients presented dyspnea an association with poor clinical outcomes was observed (HR = 7.08 (3.67; 13.64), p < 0.01). No association was found when the main complaint was abdominal pain or trauma. Detailed analyses are provided in the online Supplementary Material.

## Discussion

Our study is the first addressing real-life punctual non-palliative opioid administration to patients with COPD in an acute care setting, and not uniquely for sedation for a respiratory intervention. Opioid use was associated with an increased risk of adverse clinical outcomes in this population, including ICU admission and death. Almost all the sensitivity analyses and subgroup analyses showed a similar association between opioid drug use and poor clinical outcomes in COPD patients, except when trauma or abdominal pain were the reason for the ED visit.

An increased risk of morbidity and mortality seems to be observed in COPD patients with chronic opioid treatment^11,12^: they experience more exacerbations, pneumonia, and mortality. Cough reduction and immunosuppression may also contribute to the poor reputation of opioids in this population^19,20^. There is still debate about non-palliative opioid use by patients with COPD, hampered by a lack of data concerning the short term use of opioids, or in specific conditions (e.g. to increase comfort with NIV and as a protective effect during intubation). Significant differences in population characteristics exist between studies, in terms of age, indications, and duration of treatment. While we studied opioid administration for acute indications in an emergency setting, Vozoris *et al*. have reported on the effects of median duration (10-day) opioid regime^11^. Although we consider the same main endpoints, our study population possibly better represents COPD patients seen in an ED. Such differences between studies highlight the importance of clearly defining the scope of opioid prescription for COPD patients. Nonetheless, the results are concordant and are likely to reinforce wariness regarding non-palliative opioid use.

In contrast, several studies have stressed the value of these treatments in decreasing dyspnea, even in patients who are not terminally ill^21,22^. The study of opioid-related side-effects in COPD is often hampered by the fact that the adverse events observed are frequently the same as those occurring during the course of the disease (exacerbations, needs for ventilatory support, death etc.). This difficulty in separating the effects of disease progression itself and treatment side-effects is also complicated by limitations of care orders^23^, when opioids may have been used as a last resort and often led to death. Palliative opioid use is described in the refractory breathlessness treatment and in the management of patients with advanced COPD^24–27^. In this study, we purposely excluded patients given opioids for palliative purposes because we thought that this would mask any pejorative effect of opioids used as treatment rather than as a palliative. Some authors speak of “confounding by indication” in pharmacological studies, including COPD^28^. In the case of COPD patients consulting an ED for trauma, the risk of a limitation of care order is unlikely, and the use of opioids appeared safe. We observed a small proportion of deaths within 48 hours, and 36 patients (4.2% of patients with outcomes) died without the use of NIV, intubation or admission to the ICU. It remains possible that some of them actually underwent treatment limitation and then were given opioids for palliative care.

Another interpretation could be that of a deleterious effect of opioids for the most fragile patients, but not found in moderate cases stabilized with NIV. Theoretically opioid use could prevent NIV failures due to patient intolerance and dynamic hyperinflation phenomenon. This ventilatory physiopathology has led some authors to propose adjuvant treatments that include opioids, sometimes in titration or at low doses^12,29,30^. In our data, no patient receiving NIV and opioids required invasive ventilation, while of the 534 patients who received NIV without opioid administration, 11 (2.1%) were intubated.

The interpretation of our primary composite endpoint must be balanced by other criteria. Patients with subsequent readmission have a different risk of death at first admission, which could restrict the interpretation of our results. These patients are likely to have specific baseline characteristics, risks linked to opioids use, and outcomes of interest that are partly correlated through multiple admissions. The “frequent exacerbator” phenotype has been associated with a poor quality of life, deteriorating lung function and death^31,32^. This limitation is partly addressed by our sensitivity analysis in which we included only first admissions showing poor clinical outcomes. Analyzing only the first admission could avoid a survival bias that might otherwise be observed during subsequent readmissions.

Our study has several strengths, including a large sample size and the use of hard outcomes for safety. In the descriptive analysis we observed an increase in opioid use over time (about 20% over 5 years for COPD), and a more frequent utilization in highly severe cases as reflected by the triage level, ambulance admission, and acute pain or trauma related conditions.

However, our study has several limitations. First, Selection was based on medical records and no spirometric or radiological confirmation of the diagnosis of COPD was documented. The patient sample may therefore have included patients without COPD, who had another respiratory diagnosis. Nevertheless, the manual selection of a sample of files by a senior physician showed a good level of agreement with the automatic selection. If some patients were included in our cohort who didn’t have COPD, this might have introduced a bias, lessening the amplitude of the results and not the opposite (these patients being less at risk of poor outcomes). Moreover, the patients reported as “possible COPD” but without confirmatory test results perfectly match the profile of patients for whom the safety of opioid treatment should be considered by the emergency clinician.

Second, In this large retrospective database we were not able to collect the results of blood gas analyses for COPD patients admitted for dyspnea. Low pulse oximetry (<92%) at admission was associated with poorer outcomes and it should be kept in mind that the measurement of oxygen saturation at triage is usually made while patients are receiving oxygen treatment. In addition, we were unable to conduct a sensitivity analysis based on potent vs. weak opioids, long vs. short acting opioids and/or the posology of opioids, or to take into account associated treatments such as benzodiazepines. In patients with available co-prescription records, we observed a low prevalence of benzodiazepine use (3.7%), always administered by an enteral route and late-on in the ED stay. Moreover, in this sub-group no patient receiving both opioids and benzodiazepines experienced adverse outcome. Concerning doses, the relative low use of naloxone may suggest that clinicians were very conservative in their use of opioids. The vast majority of administrations were made at a standard analgesic posology, and few punctual small or high dosages were observed.

Third, our definition of mortality is debatable. In-hospital or 30-day mortality are more frequently used when studying clinical events or type of care. The use of opioids as studied here is very punctual and, in our opinion, it is highly unlikely that such an administration is associated with long-term death without any event occurring in the following hours, given the kinetics of this drug class.

Given the lack of data on acute care in this area, this study provides some answers, suggests caution in the use of opioids in this population and advocates for the realization of prospective controlled studies.

## Conclusions

After analysis of the data from a large retrospective multicenter cohort, we found that non-palliative short term opioid use in an acute care context was associated with poor clinical outcomes. However, given the observed variety in the use of morphine in our cohort, in various different situations and for different purposes, prospective studies are needed to assess its safety in a well-defined population.

## Supplementary information

Supplementary Material.

## Data Availability

The datasets used and/or analysed during the current study are available from the corresponding author on reasonable request.
