# Supplementary material for: Opioid drug use in emergency and adverse outcomes among patients with chronic obstructive pulmonary disease: a multicenter observational study
Source: Sci Rep. 2020 Mar 19;10:5038. doi: 10.1038/s41598-020-61887-2 (PMC7081336; doi:10.1038/s41598-020-61887-2)
Supplement: Supplementary file 1 — Supplementary Material. [file 41598_2020_61887_MOESM1_ESM.pdf]

Opioid drug use in emergency and adverse outcomes among patients with chronic obstructive pulmonary disease: a multicenter observational study

Damien Viglino[1,2*], Raoul Daoust[3]; Sebastien Bailly[2,4], Caroline Faivre-Pierret[1], Isma Charif[2], Matthieu Roustit[2,5], Jean Paquet[3], Guillaume Debaty[1], Jean-Louis Pépin[2,4], Maxime Maignan[1,2], Jean-Marc Chauny[3]

Affiliations: [1] Emergency Department and Mobile Intensive Care Unit, Grenoble Alpes University Hospital, Grenoble, France. [2] INSERM U1042, HP2 Laboratory, Grenoble-Alpes University, Grenoble, France. [3]Department of Emergency Medicine, Research Centre, Sacré-Coeur Hospital of Montreal, Montreal, Quebec, Canada. [4] Department of Physiology and Sleep, Grenoble Alpes University Hospital, Grenoble, France. [5] Clinical Pharmacology Department, INSERM CIC1406, Grenoble Alpes University Hospital, Grenoble, France.

Supplementary data

Management of the missing values

Two variables presented missing value: triage level (10.6%) and means of transport (0.1%). To account for missing values, multiple imputations were performed using a fully conditional specification method with logistic regression for categorical variables and linear regression for continuous variables. Twenty imputed datasets were created to consider variables with less than 20% of missing values. Twenty-one iterations were performed for the iteration process model and the following variables were introduced in the FCS models: the composite outcome, age, sex, year, centre, opioid administration, visual analogic scale for pain and occupational rate.

A variable selection process using a stepwise method was performed within each of the twenty imputed dataset and converged to the same variable selection. The final model was performed by using the same variable for twenty datasets, and finally results were gathered into one final model by applying Rubin's rules with the MIANALYZE procedure in SAS.

A. Separate analysis of outcomes

a. Intensive care unit admission

Variable	Class	Multivariate HR (IC95%CI)	p value
Age (years)	<56	ref.	ref.
	56-65	0.75 (0.41 ; 1.39)	0.37
	65-80	0.49 (0.29 ; 0.84)	<0.0001
	>=80	0.21 (0.11 ; 0.37)	<0.0001
Year of inclusion	2008	0.84 (0.39 ; 1.81)	0.66
	2009	0.62 (0.29 ; 1.34)	0.22
	2010	0.75 (0.37 ; 1.53)	0.43
	2011	0.88 (0.46 ; 1.71)	0.71
	2012	1.06 (0.58 ; 1.94)	0.85
	2013	1.87 (1.07 ; 3.25)	0.03
	2014	ref.	ref.
Center of inclusion	Montreal	ref.	ref.
	Grenoble	2.18 (1.44 ; 3.31)	<0.0001
Pulse oximetry (%)	<92	1.01 (0.64 ; 1.59)	0.96
Means of transport	ambulance	2.77 (1.69 ; 4.55)	<0.0001
	no ambulance	ref.	ref.
Triage level	1or 2	3.24 (2.24 ; 4.69)	<0.0001
	3 or 4 or 5	ref.	ref.
**Opioid treatment**	**(yes)**	**1.81 (1.23 ; 2.67)**	**<0.0001**

b. ***Non-invasive ventilation***

Variable	Class	Multivariate HR (IC95%)	p value
Age (years)	<56	ref.	ref.
	56-65	2.02 (1.29 ; 3.16)	<0.0001
	65-80	1.50 (0.98 ; 2.28)	0.06
	>=80	0.93 (0.61 ; 1.43)	0.76
Year of inclusion	2008	0.16 (0.10 ; 0.27)	<0.0001
	2009	0.20 (0.13 ; 0.30)	<0.0001
	2010	0.20 (0.13 ; 0.30)	<0.0001
	2011	0.74 (0.57 ; 0.97)	0.03
	2012	1 (0.79 ; 1.27)	1.00
	2013	0.98 (0.77 ; 1.25)	0.87
	2014	ref.	ref.
Center of inclusion	Montreal	ref.	ref.
	Grenoble	0.24 (0.17 ; 0.35)	<0.0001
Pulse oximetry (%)	<92	1.20 (0.95 ; 1.50)	0.12
Means of transport	ambulance	1.66 (1.36 ; 2.03)	<0.0001
	no ambulance	ref.	ref.
Triage level	1or 2	2.47 (2.07 ; 2.95)	<0.0001
	3 or 4 or 5	ref.	ref.
**Opioid treatment**	**(yes)**	**1.06 (0.82 ; 1.36)**	**0.65**

c. Intubation

Variable	Class	Multivariate HR (IC95%)	p value
Age (years)	<56	ref.	ref.
	56-65	1.83 (0.43 ; 7.76)	0.41
	65-80	1.23 (0.32 ; 4.75)	0.76
	>=80	0.52 (0.13 ; 2.14)	0.36
Year of inclusion	2008	0.26 (0.04 ; 1.69)	0.16
	2009	0.31 (0.07 ; 1.32)	0.11
	2010	0.54 (0.17 ; 1.79)	0.32
	2011	0.73 (0.26 ; 2.04)	0.55
	2012	0.56 (0.20 ; 1.57)	0.27
	2013	0.95 (0.38 ; 2.37)	0.91
	2014	ref.	ref.
Center of inclusion	Montreal	ref.	ref.
	Grenoble	2.53 (1.27 ; 5.05)	<0.0001
Pulse oximetry (%)	<92	0.68 (0.24 ; 1.91)	0.47
Means of transport	ambulance	2.07 (0.87 ; 4.96)	0.10
	no ambulance	ref.	ref.
Triage level	1or 2	2.14 (1.10 ; 4.16)	0.02
	3 or 4 or 5	ref.	ref.
**Opioid treatment**	**(yes)**	**3.41 (1.66 ; 7.01)**	**<0.0001**

d. ***Death***

Variable	Class	Multivariate HR (IC95%)	p value
Age (years)	<56	ref.	ref.
	>=80	2.41 (1.30 ; 4.47)	<0.0001
Year of inclusion	2008	1.02 (0.24 ; 4.31)	0.98
	2009	1.34 (0.43 ; 4.19)	0.61
	2010	1.45 (0.49 ; 4.27)	0.50
	2011	1.39 (0.49 ; 4)	0.54
	2012	0.89 (0.30 ; 2.65)	0.84
	2013	0.72 (0.23 ; 2.29)	0.58
	2014	ref.	ref.
Center of inclusion	Montreal	ref.	ref.
	Grenoble	3.29 (1.70 ; 6.37)	**<0.0001**
Pulse oximetry (%)	<92	1.34 (0.63 ; 2.86)	0.45
Means of transport	ambulance	0.73 (0.36 ; 1.45)	0.37
	no ambulance	ref.	ref.
Triage level	1or 2	2.04 (1.06 ; 3.94)	0.03
	3 or 4 or 5	ref.	ref.
**Opioid treatment**	**(yes)**	**5.09 (2.81 ; 9.23)**	**<0.0001**

B. Analysis by Main complaint

a. Dyspnea as main complaint (composite outcome)

Variable	Class	Multivariate HR (IC95%)	p value
Age (years)	<56	ref.	ref.
	56-65	0.59 (0.23 ; 1.51)	0.27
	65-80	0.38 (0.13 ; 1.14)	0.08
	>=80	0.71 (0.32 ; 1.59)	0.41
Year of inclusion	2008	1.16 (0.29 ; 4.60)	0.83
	2009	1.01 (0.27 ; 3.80)	0.99
	2010	1.41 (0.40 ; 4.91)	0.59
	2011	2.25 (0.72 ; 7.06)	0.16
	2012	1.53 (0.48 ; 4.91)	0.47
	2013	0.77 (0.20 ; 2.98)	0.70
	2014	ref.	ref.
Center of inclusion	Montreal	ref.	ref.
	Grenoble	4.85 (2.44 ; 9.66)	<0.0001
Pulse oximetry (%)	<92	1.08 (0.52 ; 2.24)	0.84
Means of transport	ambulance	1.75 (0.78 ; 3.94)	0.18
	no ambulance	ref.	ref.
Triage level	1or 2	2.94 (1.53 ; 5.65)	<0.0001
	3 or 4 or 5	ref.	ref.
**Opioid treatment**	**(yes)**	**7.08 (3.67 ; 13.64)**	**<0.0001**

b. Abdominal pain as main complaint (composite outcome)

Variable	Class	Multivariate HR (IC95%)	p value
Center of inclusion	Montreal	ref.	ref.
	Grenoble	4.53 (1.51 ; 13.63)	<0.0001
Pulse oximetry (%)	<92	1.58 (0.52 ; 4.85)	0.42
**Opioid treatment**	**(yes)**	**2.05 (0.8 ; 5.26)**	**0.14**

c. Trauma as main complaint (composite outcome)

Variable	Class	Multivariate RH (IC95%)	p value
Year of inclusion	2009	0.28 (0.01 ; 8.25)	0.46
	2010	0.56 (0.03 ; 9.28)	0.69
	2012	0.22 (0.01 ; 4.90)	0.34
	2013	1.21 (0.20 ; 7.36)	0.83
	2014	ref.	ref.
Pulse oximetry (%)	<92	0.45 (0.05 ; 3.66)	0.45
Triage level	1or 2	8.37 (1.67 ; 41.93)	<0.0001
	3 or 4 or 5	ref.	ref.
**Opioid treatment**	**(yes)**	**2.83 (0.54 ; 14.83)**	**0.22**

C. Sensitivity analyses

a. ***Multivariate analysis taking into account only opioids administered by a parenteral route (composite outcome)***

Variable	Class	Multivariate HR (IC95%)	p value
Age (years)	<75	ref.	ref.
	75-78	0.37 (0.10 ; 1.40)	0.15
	78-82	0.75 (0.36 ; 1.54)	0.43
	>=82	0.79 (0.47 ; 1.35)	0.39
Year of inclusion	2008	0.95 (0.28 ; 3.19)	0.93
	2009	1.29 (0.48 ; 3.44)	0.62
	2010	0.92 (0.33 ; 2.59)	0.88
	2011	1.71 (0.71 ; 4.10)	0.23
	2012	1.22 (0.51 ; 2.94)	0.65
	2013	1.27 (0.53 ; 3.06)	0.59
	2014	ref.	ref.
Center of inclusion	Montreal	ref.	ref.
	Grenoble	1.87 (1 ; 3.51)	0.05
Pulse oximetry (%)	<92	1.07 (0.53 ; 2.13)	0.86
Means of transport	ambulance	1.55 (0.82 ; 2.93)	0.17
	no ambulance	ref.	ref.
Triage level	1or 2	1.55 (0.93 ; 2.60)	0.09
	3 or 4 or 5	ref.	ref.
Main complaint	dyspnea	1.04 (0.48 ; 2.29)	0.91
	abdominal pain	1.35 (0.58 ; 3.14)	0.48
	trauma	ref.	ref.
**Opioid treatment**	**(yes)**	**5.61 (3.25 ; 9.67)**	**<0.0001**

b. Multivariate analysis taking into account only opioids administered by an oral route. Patients receiving parenteral opioids were excluded. (composite outcome)

Variable	Class	Multivariate HR (IC95%)	p value
Age (years)	<75	ref.	ref.
	75-78	0.92 (0.33 ; 2.57)	0.87
	78-82	0.91 (0.39 ; 2.11)	0.82
	>=82	0.89 (0.47 ; 1.70)	0.73
Year of inclusion	2008	0.64 (0.14 ; 2.81)	0.55
	2009	0.97 (0.33 ; 2.87)	0.95
	2010	1.25 (0.45 ; 3.47)	0.67
	2011	1.29 (0.48 ; 3.41)	0.61
	2012	1.02 (0.38 ; 2.74)	0.97
	2013	0.73 (0.25 ; 2.11)	0.56
	2014	ref.	ref.
Center of inclusion	Montreal	ref.	ref.
	Grenoble	2.65 (1.31 ; 5.38)	<0.01
Pulse oximetry (%)	<92	0.70 (0.37 ; 1.32)	0.27
Means of transport	ambulance	1.25 (0.62 ; 2.49)	0.53
	no ambulance	ref.	ref.
Triage level	1or 2	2.04 (1.14 ; 3.64)	0.02
	3 or 4 or 5	ref.	ref.
Main complaint	dyspnea	0.81 (0.33 ; 1.96)	0.64
	abdominal pain	1.24 (0.48 ; 3.24)	0.66
	trauma	ref.	ref.
**Opioid treatment**	**(yes)**	**5.95 (2.60 ; 13.58)**	**<0.01**
